# Simulation-based learning in teacher education: Using Maslow’s Hierarchy of needs to conceptualize instructors’ needs

**DOI:** 10.3389/fpsyg.2023.1149576

**Published:** 2023-04-06

**Authors:** Rivi Frei-Landau, Orna Levin

**Affiliations:** ^1^The School of Education, Achva Academic College, Shikmim, Israel; ^2^The Department of Education and Psychology, the Open University, Ra’anna, Israel

**Keywords:** simulation-based learning, teacher education, instruction, Maslow’s Hierarchy of needs, qualitative case study

## Abstract

**Introduction:**

Simulation-based learning (SBL) has become an effective tool in the education field, and instructors play a significant role in leading it. Although much is known about participants’ needs, SBL instructors’ needs have yet to be addressed. The study’s goal was to explore SBL instructors’ needs while guiding an SBL workshop using the psychological framework of Maslow’s Hierarchy of Needs.

**Methods:**

We employed a qualitative case-study design, consisting of 68 four-hour-long workshops, held at a teacher-education simulation center by the center’s professional instructors. Data collection comprised 211 statements derived from instructors’ open-ended reflections, the transcripts of two focus groups held with the instructors, and 98 interpersonal communication documents.

**Results:**

Data were analyzed using both deductive and inductive thematic analysis, which rendered 11 themes spanning Maslow’s five levels, and revealing two possible simulation-based learning paths: a complete process, in which all needs are met and an incomplete process, in which needs remain unmet.

**Discussion:**

Theoretical insights and practical implications are provided for attending to instructors’ needs (i.e., basic needs, security, belonging, self-esteem, self-actualization) to ensure optimal learning in teacher education when using SBL.

## 1. Introduction

The use of simulation-based learning (SBL) in teacher education has increased rapidly over the last decade (Ferguson, 2017; Thisgaard and Makransky, 2017; Ledger et al., 2022), and SBL instructors play a significant role in leading it. Although much is known about workshop participants’ needs, instructors’ needs have yet to be addressed, particularly in the field of teacher education. This lack of knowledge may result in a situation in which instructor’s needs are overlooked, thus, impeding their ability to perform optimally. Hence, to addresses this literature gap, the goal of the current study was to explore instructors’ needs when leading an SBL workshop and to identify and conceptualize them using Maslow’s Hierarchy of Needs, a well-established framework regarding human needs. Enhancing our theoretical understanding of the understudied phenomenon of instructors’ needs is imperative, given their significant role in facilitating the workshop goals; thus, addressing their needs is a key factor for the successful implementation of SBL in teacher education. This view coincides with previous claims (e.g., Snoek et al., 2010) that identify the teacher educator as the figure with the greatest impact on the quality of teacher education.

### 1.1. Theoretical background

#### 1.1.1. Simulation-based learning

Simulation-based learning is a novel methodology that evolved from health disciplines (Zhong and Zhang, 2021), and authentically simulates the conditions of the professional arena at hand for learning purposes such as teaching skills (Chernikova et al., 2020; Ross and Atkinson, 2020; Malone and Brünken, 2021; Frei-Landau et al., 2022; Muchnik-Rozanov et al., 2022). It is accompanied by a reflective debriefing (Garden et al., 2015) and guided by an instructor, during which time constructive feedback is given, with the aim of improving future performance (Girod and Girod, 2008).

In its early stages, the term ‘simulation’ was used to describe role playing, whereas over the last two decades it has been expanded to include advanced designs that involve professional actors and the latest technology (Frei-Landau and Levin, 2022). For instance, *computer-based* simulations, such as augmented-reality environments use video devices that imitate the physical world (Enyedy et al., 2015; Tang et al., 2021) and *human* simulations - often termed ‘clinical simulations,’ involve professional actors (Dotger, 2010). Despite the diversity of SBL designs, they share several common features: learning in a safe environment but under conditions that resemble those of the professional field in question (Rayner and Fluck, 2014), learning from mistakes under conditions that involve no risk or deleterious impact (Kaufman and Ireland, 2016), and learning through active and reflective experience (Butvilofsky et al., 2012).

#### 1.1.2. Simulations in the field of teacher education

Until recently, SBL has been used mostly in medical and health education (Fischetti et al., 2021). Although its use in the field of teacher education is still in development (Shapira-Lishchinsky, 2013; Levin and Flavian, 2020; Frei-Landau et al., 2022), it has been purported to help teacher-education graduates to be better prepared for facing future challenges (Dieker et al., 2014; Kaufman and Ireland, 2016), by providing an opportunity to apply theoretical knowledge to behavior (Theelen et al., 2019). As such, simulations are recommended and implemented in initial teacher education (Ledger and Fischetti, 2020; Ledger, 2021; Ade-Ojo et al., 2022), and recent reviews have demonstrated SBL’s pedagogical benefits, specifically, promoting cognitive, behavioral, and affective learning processes (Vlachopoulos and Makri, 2017).

Theoretically speaking, learning through simulations is grounded in sociocultural frameworks for embodied cognition (Danish et al., 2020). Specifically, according to Vygotsky’s Zone of Proximal Development (ZPD in Vygotsky, 1978), learners acquire skills *via* guidance and encouragement of a more skilled partner–in the case of SBL–the instructor. In fact, the SBL instructor guides a reflective debriefing during the learning process. This is in line with Dewey’s suggestion that educators undertake reflective practices for self-development, as well as improved self-confidence and self-efficacy. To this end, current simulation studies rely on these theoretical frameworks (Fischetti et al., 2021).

Studies focusing on SBL outcomes have demonstrated the affordances of SBL in the field of teacher education, which include the acquisition of skills specific to teacher education (Theelen et al., 2019) and special education (Larson et al., 2020); the opportunity to formulate cognitive knowledge (Ferguson, 2017; Dalinger et al., 2020); and the promotion of learners’ reflective abilities (Levin and Flavian, 2020) and critical thinking (Codreanu et al., 2020) in routine practice, as well as in the context of ethical dilemmas in education (Yablon et al., 2021). However, it is important to note that studies have also indicated that there are also drawbacks to SBL. For instance, in the course of the simulation, learners can experience negative feelings, such as embarrassment and demotivation (Bautista and Boone, 2015; Dalgarno et al., 2016), as well as learning overload (Makransky et al., 2019), each of which might hinder the learning process. Given these findings, the efficacy of SBL in the field of teacher education requires further study. Interestingly, SBL advantages and disadvantages have been examined from participants’ perspectives but rarely from those of instructors (Judge et al., 2013).

### 1.2. The significant role of the instructor in simulation workshops

As the reflective debriefing phase is the most significant component of SBL (Tutticci et al., 2018) and given that the instructors are the ones to lead it, their needs should be examined. Furthermore, as the quality of instruction affects the ultimate efficacy of SBL (Garden et al., 2015), providing instructors with optimal conditions is imperative. In this sense, the instructor’s role coincides with the teacher educator’s role (i.e., guiding the learner through the learning process). Hence, in accordance with prior arguments that the teacher educator is the figure with the greatest impact on the quality of teacher education (Snoek et al., 2010), SBL instructors’ needs should be examined. In other words, acknowledging that it was important to study the needs of teacher educators in the field of teacher education (Van Velzen et al., 2010; Ghousseini, 2017) given the critical role they play (Orland-Barak, 2014), we must conclude that it is equally important to address the needs of simulation instructors in the context of SBL in teacher education.

The literature in the field of medical simulations indicates that the instructors deal with complex challenges, such as creating a supportive group climate (Tutticci et al., 2018) and managing the technical operation of equipment (Sellberg, 2018), especially the video-system equipment. By contrast, the challenges and needs of simulation instructors in the field of teacher education have yet to be precisely identified and mapped. This gap in the literature is surprising, considering the vital role of instructors during SBL. Therefore, expanding our understanding of instructors’ needs by mapping them is expected to provide insight into ways to optimize the instructors’ functioning (Der-Sahakian et al., 2015; Campbell and Daley, 2017). In the current study, we addressed this overlooked aspect, by examining SBL instructors’ needs through the lens of Maslow’s Hierarchy of Needs (Maslow, 1943).

#### 1.2.1. The theoretical framework: Maslow’s Hierarchy of needs

Maslow’s Hierarchy of Needs (MHN) deals with the essence of human needs and motivations (Fives and Mills, 2016) and provides a hierarchical scale onto which such needs can be mapped. According to Maslow, this hierarchy ranges from basic to complex needs, as follows: (1) *Basic needs* include physiological needs such as hunger, thirst, and sleep, the fulfilment of which are necessary for survival. From the perspective of scholars in the field of education (Riley and Mort, 1981; Fisher and Royster, 2016), the equivalent of this level of basic need in the context of the education field also includes tangible equipment and supplies as well as technology-related needs (Bailey and Pownell, 1998); (2) *Safety needs* involve a sense of security and protection from physical as well as emotional harm (freedom from threat and the need for self-preservation); (3) *Belongingness needs* involve the desire for acceptance, affection, and friendship, as well as the ability to exist in harmony with others; (4) *Esteem needs* consist of two elements: the need to feel competent, strong, and successful, and the need for recognition, appreciation, and a positive reputation (Maslow, 1943 in Adams et al., 2015); and (5) *Self-actualization needs* include the realization of one’s goals/ambitions (Shaughnessy et al., 2018), and are gratified when individuals reach their potential.

Maslow’s main claim was that ‘The appearance of one need usually rests on the prior satisfaction of another’ (Maslow, 1943, p. 370). Hence, the satisfaction of basic existential needs is a preliminary condition for engaging in processes of more complex growth (such as learning). It may be for this reason that Maslow’s theory is used so extensively in the field of education (Fives and Mills, 2016).

Maslow’s theory reached the height of its popularity in the 1960s and 70s. From the end of the 1960s to the mid-1980s, numerous studies applied the theory to gain an understanding of candidates’ motivations for pursuing a career in the field of education (Coon, 2006), the needs of preservice teachers when learning, and the desired components of teacher-education programs (Riley and Mort, 1981). Between the mid-1980s and the end of the 1990s, the theory was rarely referenced in studies.

In the last two decades, there has been a renewed interest in Maslow’s theory, with a focus on contemporary issues. Maslow’s theory has been found to be a suitable framework for understanding schoolteachers’ needs (Fisher and Royster, 2016), as well as scholars’ needs in higher education (Schulte, 2018). Furthermore, in the field of teacher education, it was used for conceptualizing instructors’ process of self-actualization through mentoring (Fletcher, 2006). Thus, in line with these developments, as well as the findings of a preliminary pilot study that highlighted MHN as a relevant theoretical framework for mapping instructors’ needs (see the data analysis section), we chose to use MHN as a theoretical lens for exploring the overlooked issue of SBL instructors’ needs. Surprisingly, no studies about simulation instructors’ needs exist despite the centrality of instructors in the SBL process as the debriefing facilitator. Providing insight into SBL instructors’ needs is crucial, as meeting people’s needs is linked with their success (Freitas and Leonard, 2011). In conclusion, considering the lack of empirical information regarding the needs of SBL instructors’ in teacher education, and the importance of this information given the instructors’ central role in facilitating the workshop goals, the current study aimed to explore SBL instructors’ needs in the teacher education arena.

### 1.3. Research questions

As mentioned, not only is it crucial to identify SBL instructors’ needs, but once identified, we must be prepared to interpret and evaluate their impact, to understand their relative significance to the overall SBL process. To this end, we formulated the following research questions:

1. Instructors’ perceived needs and their mapping according to MHN: what are the needs of simulation instructors when leading a simulation workshop, and in what ways do these needs correspond to Maslow’s Hierarchy of Needs theory?
2. Instructors’ perceived SBL process in light of their needs’ gratification: What is the perceived impact of gratification of the needs in the hierarchy vis-à-vis the SBL process overall? What is the perceived impact of unmet needs on the SBL process?

## 2. Methodology

### 2.1. The study context

The research was conducted at a simulation center at a higher-education institution in Israel that operates in accordance with the clinical simulation model. This model involves professional actors (as opposed to virtual simulations) and is frequently used by Dotger (2015) to enhance teacher preparation. Generally, workshops are directed toward promoting communication skills, teamwork, and multiculturalism (Yablon et al., 2021; Frei-Landau and Avidov-Ungar, 2022; Levin et al., 2022).

The center is located in the school of teacher education and its content is coordinated with the teacher education program. It provides services to both preservice and inservice teachers in separate groups. Inservice teachers participate in SBL as part of their professional development courses, whereas preservice teachers typically participate in SBL as part of a course in pedagogy. The students attending the center represent a multicultural cross-section of Israeli society, which includes Israeli Jewish students (secular and religious alike), Israeli Arab students (mostly from local Bedouin settlements), and new immigrants.

The current study was carried out over 2 years. The staff of the simulation center consists of 29 instructors and 14 professional actors. Workshops are guided by a single instructor, who has received special training for this purpose. Most of the instructors are the college lecturers, whereas paucity hold a background and contemporary employment that is highly related to teacher education. Specifically, most of them are the college’s pedagogical teacher educators, whereas some are senior schoolteachers or hold a managerial position in their school. In addition to the specialized training they receive at the simulation center, they usually hold prior additional certifications as group mentors or coachers; thus, they have education-oriented backgrounds as well as field experience leading groups. The simulation center’s staff, including the simulation instructors, the center’s administrators and the research unit meet regularly to enable knowledge sharing and brainstorming on how to best design and improve the quality of the teacher-education simulations. Each simulation workshop lasts 4 h and comprises approximately 12–15 participants. Pre-workshop preparation includes an in-depth conversation held between the group’s coordinator and a center staff member, during which the main needs of the group are identified. Next, the instructor receives additional information about the incoming group’s characteristics; then, tailored scenarios are written by the center’s professional content developers (scenario writers). Each workshop consists of three different scenarios typically involving conflictual situations that are common in the field of education. Table 1 illustrates a sample of simulation scenarios, presenting the contexts, descriptions, goals, and key issues for instructors’ debriefing.

**Table 1:** A sample of simulation scenarios: Contexts, descriptions, goals, and key issues for instructor’s debriefing.

Simulated context	Scenario’s figure characters	Scenario description	Summary of key issues for instructor’s debriefing
Teacher*-student	Dan, 13 years old, diagnosed with ADHD	Teacher-initiated conference to address student’s classroom disruptive behaviors (chattering, inattention, etc.) and learning difficulties	Facilitating cooperation Finding empathy Evoking motivation for learning Assertiveness
	Sara, 14 years old, has recently entered junior high school	Teacher-initiated meeting to discuss student’s social behaviors (social-media shaming)	Active listening Inquiring through open questions Expressing empathy Assertiveness
Teacher*-parent	Lea, 45 years old, a lawyer, mother of a 10-year-old student diagnosed with ASD	Parent-initiated conference to discuss with the teacher the problems of the inclusion process of her fifth-grade daughter with autism	Facilitating trust Legitimizing emotions Dealing with antagonism Collaborative problem-solving
	Jacob, 55 years old, a delivery man, father of a nine-year-old student	Teacher-initiated conference to discuss student’s off-task behaviors and low grades, aiming to suggest professional assessment	Facilitating trust Managing goal-oriented conversation Managing resistance Legitimizing emotions
School principal – teacher*	Kim, an experienced school principal, a strong believer in parent-school cooperation	School-principal-initiated conference to acquaint the teacher with the school’s educational philosophy, procedures, and policies, following a parent’s complaint	Accepting criticism Discussing expectations Facing an authority figure Managing impressions
	Alex, an experienced school principal, highly ambitious and eager to prove himself	Teacher-initiated conference to discuss the teacher’s overload with extra school assignments	Assertiveness alongside empathy Exhibiting problem-based attitude (rather than emotion-based attitude) Managing a collaborative dialogue

* The volunteer participant.

**Figure 1:**
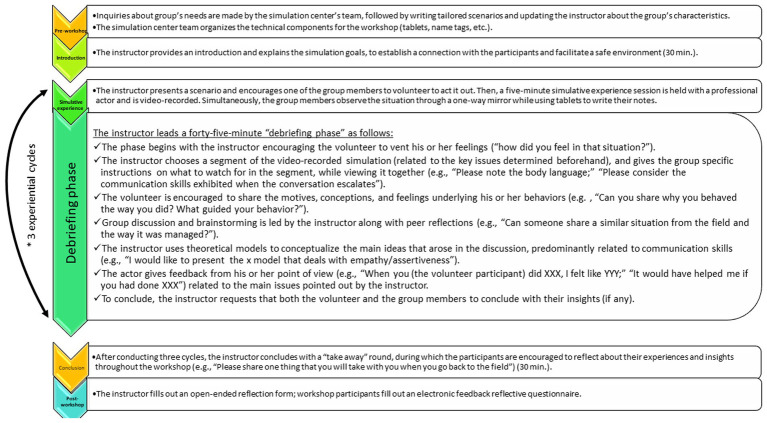
Instructor’s role throughout the SBL workshop. A step-by-step description of the simulation workshop process, emphasizing the role of the instructor throughout the process. [Note that although all workshops have the same structure, they differ in terms of their contents (different scenarios)].

The SBL workshop process involves three experiential cycles that follow the same procedure but are diverse in terms of content. Each of these cycles includes a scenario-based simulative experience – that is, a five-minute enactment of the scenario by professional actors interacting with a group member in real-time, during which the actor responds according to predefined schemes written into the scenario – followed by a debriefing phase. The interactions are video-recorded (using the SimBoost platform), with the assistance of a technical support team, and the debriefing phase is conducted by the instructor, during which a variety of theoretical coping-strategy models are presented. Finally, as part of the debriefing process, the actors present their feedback to the workshop participants, providing the participants with a unique opportunity to receive direct and authentic feedback on how their behavior was experienced by others. Figure 1 presents a step-by-step description of the simulation workshop process, emphasizing the role of the instructor as well as the stages of the debriefing phase, as these are major components of SBL.

### 2.2. The study design

Gaining insight into the subjective needs of instructors called for a qualitative methodology (Creswell, 2007). In this domain, a qualitative case-study design was selected (Stake, 2013). A case-study involves the exploration of ‘a real-life, contemporary bounded system (a case), through detailed, in-depth data collection (Creswell, 2007, p. 97). In the current study, all 68 SBL workshops were conducted at a single site (the simulation center) and all dealt with promoting communication skills within conflictual situations that are typically inherent to the teaching experience. Hence, the SBL workshop can be considered a case through which instructors’ needs could be examined. The goal of this approach was to perform an in-depth case analysis of instructors’ needs while they were managing an SBL workshop. Thus, the identical structure of the workshop, which was used consistently to train teachers (albeit of different disciplines and varying levels of experience) served as an optimal setting for thoroughly exploring SBL instructors’ needs. As per Creswell’s (2007) guidelines for case-studies, we gathered a variety of data using several information sources, to provide an in-depth view of the case, which was limited in terms of time and or space.

### 2.3. Participants and data collection

Employing a purposive sampling method, 29 simulation center instructors were approached, yet it was emphasized that participation was voluntary. Eventually, 22 instructors agreed to participate. Data from 68 workshops were collected over a two-year period (28 from the first year; 40 from the second year), using multiple data sources, to provide a comprehensive understanding of the issue, allow for the triangulation of findings (Bogdan and Biklen, 2007), and the performance of cross-validity checks (Creswell, 2007). The following data sources were used.

1. *Instructors’ post-workshop reflections.* Following each workshop, instructors were asked to freely elaborate about their experiences during the simulation workshop (including perceptions, challenges, etc.). Overall, 68 reflections were collected, yielding 211 statements.
2. *Focus groups.* Two focus groups were conducted at the end of each year (10 participated in the first-year focus group and 12 participated in the second-year focus group). During the focus groups, the participants were requested to discuss their needs when leading a workshop as well as to respond to others’ comments (sample questions: ‘We wish to learn more about your needs as instructors when leading a simulation workshop. Could you please share your experience in this regard?’; ‘Please describe what promotes or impedes your needs being met in the context of leading an SBL workshop?’; ‘How does having your needs met affect the learning process and workshop overall?’, etc.). Focus group sessions lasted one and a half hours and were audio-taped and then transcribed.
3. *Instructors’ email correspondence documents.* Documents containing email correspondence between the center’s academic manager and the 22 instructors were collected. Overall, 98 documents were reviewed.

The rationale for collecting data from multiple sources lies in the assumption that richer information will be derived this way. The inclusion of personal reflections are recorded privately and covey thoughts and emotions that might not be shared in a group. By contrast, focus groups are conducted collaboratively and publicly, where interpersonal interactions can trigger new insights and perspectives. The rationale for examining email correspondences between instructors and academic managers was that this medium is often used by instructors to discuss challenges and experiences following an SBL workshop, thus, providing important data about the instructor’s needs.

Prior to data analysis, all data were coded as follows: instructors’ reflections were coded sequentially (REF-W1—REF-W68); focus group data were coded in accordance with the year in which they were conducted (FOGR1, FOGR2); and instructors’ correspondence documents were coded chronologically (ECO1—ECO98).

### 2.4. Data analysis

Data from a preliminary pilot study of simulation instructors (comprising a focus group, N = 8; and 26 email correspondences) revealed that the instructors’ needs could be mapped to the MHN framework. Thus, the MHN framework was chosen as the theoretical lens through which the entire data were analyzed and through which the issue of needs was examined. This analysis was conducted in a two-step thematic analysis procedure, as follows. In the first round, we performed a deductive analysis, using MHN as a theoretical framework through which to investigate the perceived needs of the instructors when leading simulation workshops. At this stage, data from all sources were coded based on Maslow’s five levels of human needs, while we remained open to additional levels or needs not mentioned in MHN. We calculated the frequency with which each theme appeared in each of Maslow’s levels, across all data sources, to gain a sense of the prevalence of the various themes. During the counting procedure, we took note of instances where the same instructor mentioned the same workshop-related theme in different data sources. In such cases, the theme in question was counted only once.

In the second round, we employed an open thematic analysis, which is inductive in nature. During this inductive analytic process, data segments that were identified in the previous round as reflecting instructors’ needs were analyzed, as we searched for salient themes emerging from the text. Similar or related themes were grouped together. This microanalysis was used to ensure that no important ideas, themes, or constructs were overlooked. This process yielded 11 main themes along Maslow’s five levels. Eventually, we were able to organize the emergent themes that represented instructors’ needs according to MHN. Then, we calculated the frequency with which each theme appeared in each of Maslow’s levels across all data sources, to gain a sense of the prevalence of the various themes. During the counting procedure, we took note of instances where the same instructor found a particular workshop-related theme in more than one data source (e.g., in the reflections and in the email correspondence). In such cases, the theme in question was counted only once. Table 2 presents an example of the qualitative content analysis coding process.

**Table 2:** A demonstration of the coding process using qualitative content analysis: An example of instructors’ needs that correspond to MHN-level 1, Basic Needs.

MHN level	Themes	Description of need	Sub-themes	Unit of Analysis (Examples of coded data)
Basic needs (level 1)	Equipment	Initial needs that enable the instructor to operate the workshop appropriately	Satisfactory technical equipment	“The binders with the forms were not well-organized. I had to search for the correct form…” (REF-W31) “The name badges should have been prepared beforehand. I had to waste a lot of time searching for these stickers instead of focusing on what really matters” (REF-W4)
	Workshop Ingredients	Initial needs that relate to the simulation’s unique auxiliary components	Video system facilities	“Because of technical problems in the video system, I was unable to use it throughout the debriefing stage. It complicated everything for me” (ECO2) “Observing the recorded video segment is one of the most important things that help me to guide the group towards the points I wish to teach during the workshop” (REF-W18)
			Adjusted scenario	“The scenarios were so precise and adjusted to the group – in a way that it seemed to imitate what they were dealing with in real life; the debriefing went quite smoothly. I think this was one of my best simulation workshops!” (REF-W44)
	The Workshop Actors	The need for effective acting as well as collaboration between the actor and the instructor	Quality of actor’s performance	“The actor did not do a professional acting job and it made the debriefing very challenging. In contrast, in last week’s workshop, the actor did a good job acting, and it really made a difference when I guided the debriefing” (REF-W9)
			Actor’s feedback	“Generally, the actor to is able to deliver a complex message since it is based on his or her own experience during the simulation. This is all the more evident when the actor listens to the discussion held and internalizes the key points I am striving for, and then accommodates the feedback to these key points” (FOGR2) “The actor and I took a few minutes to consult and coordinate: ‘I will focus on this and you’ll focus on that.’ This way, the students do not get bored and I know what I want to focus on” (REF-W33)

Working separately, we (the two researchers) initially read and analyzed the material, marking and coding the relevant themes and subthemes, and then ascribed each of these themes to one of the five levels of MHN. Subsequently, we compared our findings and discussed them until we reached a joint decision regarding the relevant themes at each level. Finally, member checking, which is considered the gold standard for establishing trustworthiness (Kornbluh, 2015), was employed, by sharing the findings with the instructors and incorporating their suggestions into our interpretations.

### 2.5. Procedure and ethics

This study’s protocol was approved by the higher-education institution’s Ethics Committee. The participants gave their informed consent. The request that they write their reflections was sent to instructors *via* a link to a Google Form, enabling them to refuse without being inconvenienced. Confidentiality was maintained, as answers were anonymous. Before conducting the focus group, the instructors gave their consent to have the session recorded, and they were explicitly told that they could choose what they wished to share, and were free to leave at will, without consequence. Focus groups were recorded and transcribed; all personal information was concealed and, hence, pseudonyms are used herein.

## 3. Findings

First, we will describe the themes that emerged as related to instructors’ needs during SBL, while considering the ways in which these themes correspond to MHN. Then, we will focus on two possible SBL pathways that demonstrate SBL outcomes when needs are met as opposed to when they are unmet.

### 3.1. Instructors’ needs as mapped onto MHN

**Figure 2:**
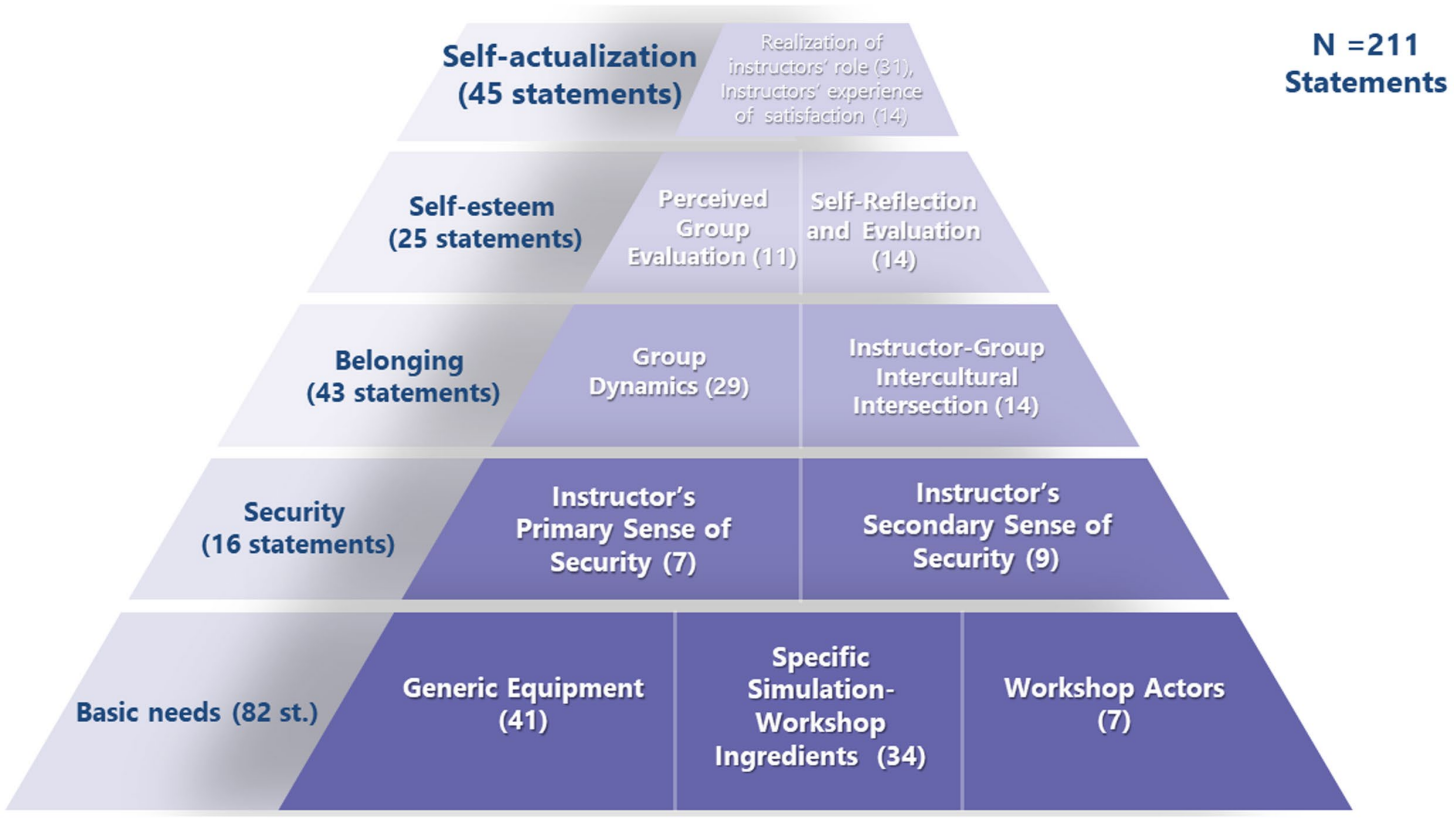
Instructors’ needs according to the MHN. Frequencies of instructors’ need-related statements arranged according to the MHN levels. In parentheses: number of statements per theme; on the left: number of need-statements per MHN level and their percentage of the total number of need-statements at all levels.

The findings unveiled the various themes that represented instructors’ needs and the ways in which these needs resembled those of MHN. Figure 2 presents the integration of these 11 themes into the five hierarchical levels.

### 3.2. Basic needs

Based on the analysis, 39% of the instructors’ reports (82 phrases) described needs that corresponded to basic needs on the MHN framework. That is, the instructors indicated that these items were essential for them to be able to perform their duties during workshops. These comprised both generic needs and specific simulation-workshop-related needs, as elaborated below.

#### 3.2.1. Generic workshop equipment

The *equipment* needed for operating the workshop was mentioned as a primary and significant concern: ‘The binders with the forms were not well-organized. I had to search for the correct form … We had to do last-minute photocopying … how am I meant to manage the workshop efficiently like this?!’ (REF-W31). The presence or absence of technical equipment was experienced as enabling or impeding the instructor’s operation of the workshop. Judging from the tone of these comments, it appears that having equipment-related difficulties was a frustrating experience.

#### 3.2.2. Specific SBL workshop components

Other basic components needed were essential to the simulation, specifically the video recording and the scenario. First, *being able to use the video* system properly during the debriefing process was experienced as essential to the instructors’ functioning: ‘Because of technical problems in the video system, I was unable to use it throughout the debriefing stage. It complicated everything for me’ (ECO2). In such cases, instructors noted that the assistance of a technical support team was beneficial.

Second, an *adequate and adapted scenario* was experienced as fundamental to instructors’ ability to successfully lead the workshop.

I once had a team of teachers who worked at a boarding school for high-risk students and had no interactions with the students’ parents. Yet the scenario focused on teacher-parent relationships. The debriefing was dull. I had to try much harder to make something out of it … in order to best function I need you to carefully select the scenario (FOCGR1).

In other words, when the scenario fails to correspond precisely to the profile of the workshop participants, the instructor’s ability to focus the debriefing phase on the predefined goals is hindered, and thus the workshop fails to address the participants’ needs.

Interestingly, the abovementioned needs were typically mentioned at the beginning of the instructors’ descriptions, and if these needs were not met, their style became laconic, and they offered no further elaborations about other, higher-level needs. This observation further suggests that these needs were fundamental to instructors’ functioning. Instructors also mentioned a human-based element as basic to their functioning: the professional actors.

#### 3.2.3. The workshop actors

According to the instructors, the participation of professional actors provided them with the optimal conditions under which to conduct the debriefing phase. Two elements were of particular importance: the *quality of the actor’s performance* and the *actor’s feedback*. Specifically, the better the actor’s performance, the more authentic the experience was for the participants, thus facilitating the instructors’ work: ‘The actor performed his role so well! It significantly helped me deliver my main message, as the situation was experienced as authentic and thus initiated behaviors that align with the predefined key issues’ (ECO88). Furthermore, the *actor’s feedback* during the debriefing helped instructors to lead the group to the predefined goals.

The actor is able to deliver a complex message since it is based on his or her own experience during the simulation. This is all the more evident when the actor listens to the discussion held and internalizes the key points I am striving for, and then accommodates the feedback to these key points (FOGR2).

In fact, some of the instructors noted that they deliberately coordinated with the actors to obtain their cooperation in the debriefing stage. Thus, it appears that the actors’ professional performance, feedback, and collaboration were perceived as essential basic ingredients for instructors’ optimal functioning.

### 3.3. Security needs

Data analysis indicated that 7.5% (16 phrases) of the instructors’ reports referred to issues of security. According to Maslow (1943), this stage refers to a sense of protection from emotional harm, as part of the need for self-preservation. Instructors’ security-related needs involved both a primary and secondary sense of security.

### 3.4. Instructors’ primary sense of security

Instructors related to their internal sense of emotional security, which stemmed predominantly from how well they were able to lead the workshop and seemed to depend on their prior professional training: ‘Unfortunately, I did not come sufficiently prepared to lead the workshop … I would have felt less threatened if, during the initial training, we would have gained more first-hand practice in leading a workshop’ (ECO12). The better instructors’ preparation was, the more they seemed to experience a primary sense of internal security in leading the workshop. Yet this sense of security was also affected by externally-based influences, as elaborated below.

### 3.5. Instructors’ secondary sense of security

Frequently, an authority figure (school principal, supervisor, etc.) attends the workshop along with the participants, a situation which can undermine the instructors’ status and emotional security: ‘The only challenge I had to cope with was the presence of the supervisor, who openly expressed her dissatisfaction with the way I was leading the workshop’ (FOGR2). Receiving criticism from authority figures during workshops may directly impact instructors’ sense of security, but their presence may also have an indirect effect on instructors’ sense of security:

It would be very helpful if the group supervisor was not present … This had a negative effect on me, as it seemed that some of the teachers felt completely blocked and the conversation was stilted … I felt their tension … They couldn’t speak openly. It made my job much harder (REF-W61).

**Figure 3:**
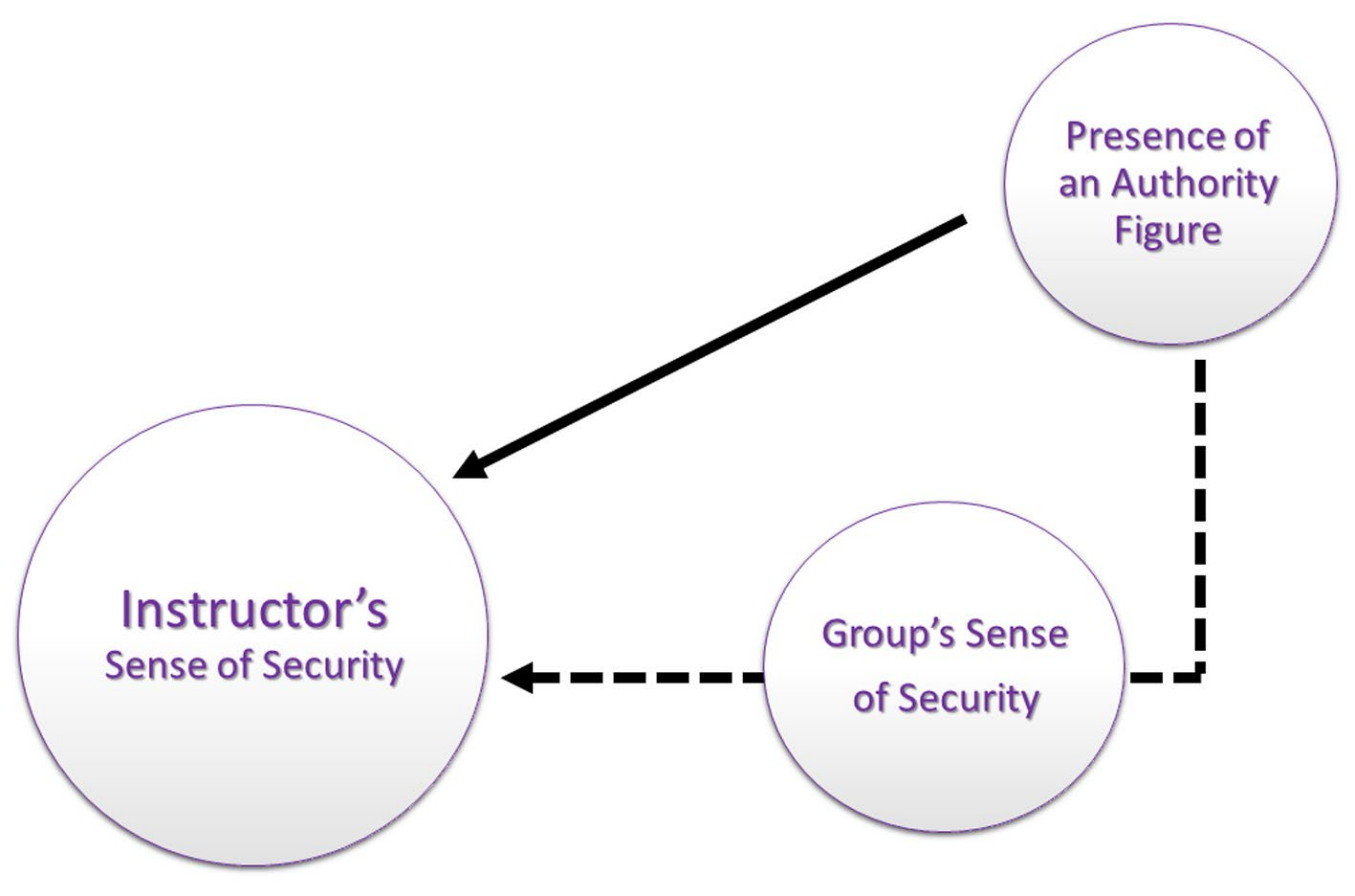
SBL instructors’ second level of MHN: Security Needs. The assumed paths thorough which the authority figure influences on the instructor’s sense of security, as emerged from the instructors’ reports of their sense of security: directly (represented by bold line) and indirectly (represented by broken line).

As shown, the authority figure may indirectly affect the instructor’s sense of security: when the participants feel threatened, they are less willing to openly engage in the debriefing process, and the instructor is thus forced to lead the workshop under challenging conditions. Figure 3 illustrates the assumed paths through which the authority figure impacts the instructor’s sense of security: directly (represented by a bold line) and indirectly (represented by a dotted line).

### 3.6. Belongingness

Forty-three phrases (20%) dealt with issues of belongingness. Within this level, two themes emerged: the first relating to ‘regular’ ongoing situations and the second being relevant in cases of group diversity.

#### 3.6.1. Instructor-group dynamics during the workshop

Instructors’ collaboration with the group during the workshop was described as crucial to their ability to create an atmosphere that promotes learning. When this collaboration was suboptimal, instructors’ functioning was hindered:

My interaction with the group was problematic. No one volunteered to participate in the simulation; they were quiet and not responsive. Some spent much of their time on their mobile phones. It was really difficult because I need to properly communicate with them to be able to function at my best (REF-W17).

Generally, instructors mentioned the need to work harmoniously with the group when guiding the workshop, for the sake of optimal operation of the workshop (recruiting a volunteer, achieving cooperation, facilitating the debriefing). This need for belongingness was potentially challenged when facing specific cases of cultural diversity.

#### 3.6.2. Instructor-group intercultural gap

Some instructors perceived the group’s cultural background as potentially challenging to their sense of belongingness. To face this challenge, they searched for information about the group beforehand, to become familiar with its practices.

I had a workshop with a religious school, and I’m glad that a few days beforehand I entered their school’s website and learned who they are and which projects they undertake. I did that because I am not religious, and their worldview is so different from my own. Later on, during the workshop, when they spoke about the project, I knew what they were talking about, so I wasn’t an outsider (FOGR1).

As shown, familiarizing themselves with the group’s cultural characteristics may help instructors to ‘work in harmony with the group’, particularly when the group’s background is unfamiliar to the instructor.

Not sharing the same native language presents another intercultural challenge. As described by a Jewish Hebrew-speaking instructor who managed an SBL workshop for a Bedouin group:

During the group discussion, they repeatedly commented to each other in Arabic, and I don’t know Arabic. If I knew Arabic, these comments could have been further developed during the discussion … and to be honest, at a certain point they were laughing and I wasn’t sure whether they were laughing about me or about the topic discussed (REF-W25).

It is worth noting that the instructor positioned herself outside to the group (*they* know … *I* do not), which precluded any sense of belonging. In such cases, the instructor’s ability to communicate and ‘join’ the group is impeded. Consequently, instructors’ ability to lead the collaborative discussions is impaired, and they may experience a sense of disconnection from the group.

### 3.7. Self-esteem needs

Findings indicated, that 12% (25 phrases) of the instructors’ reports dealt with issues related to self-esteem needs, which is in line with Maslow’s (1943) self-esteem needs.

#### 3.7.1. Instructors’ self-reflection and evaluation of functioning

Instructors’ self-esteem needs manifested first and foremost in their self-reflection about their competence and functioning: ‘I wonder how I can do it more effectively … I’m not sure whether we went deep enough’ (REF-W46). This engagement in a self-reflective process was present in many instructors’ reports and appeared to focus on instructors’ sense of competence in achieving their highest level of professional functioning. This concern aligns closely with one of the aspects (i.e., the need for competence and achievement) that comprise self-esteem needs according to Maslow (1943).

#### 3.7.2. Instructors’ perception of group evaluations

**Figure 4:**
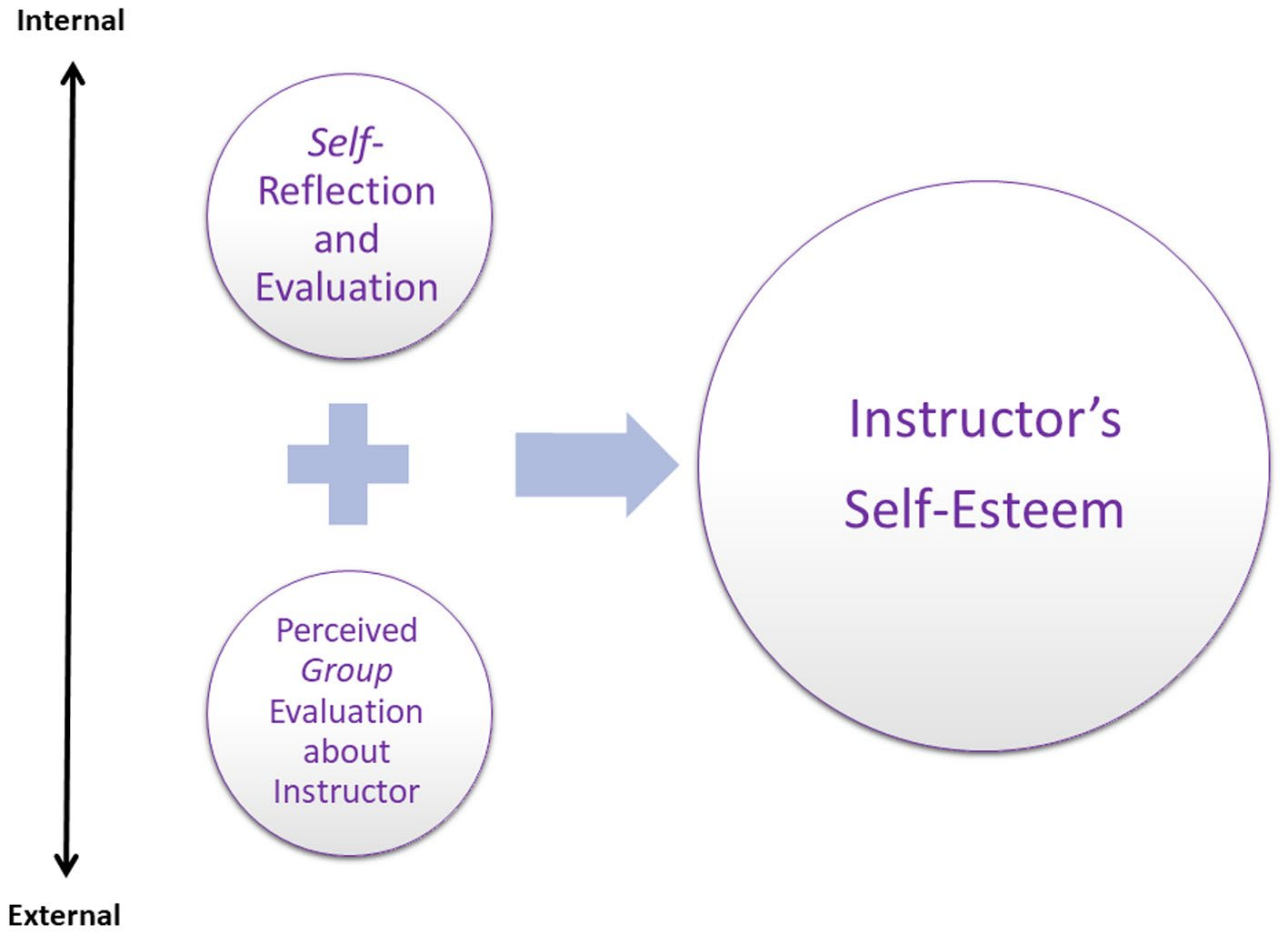
SBL instructors’ Fourth level of MHN: Self-Esteem Needs. The two sources that nurture the instructor’s self-esteem needs, as they emerged from instructors’ reports.

Another aspect of self-esteem, according to Maslow, is the need for recognition and appreciation. Indeed, positive participant responses seemed to be very important to the instructors: ‘The participants’ feedback was very warm; they said it had been a meaningful experience. They even asked to have an additional future workshop with me’ (FOGR1). The phrase ‘with me’ implies that the instructor viewed participants’ feedback as reflecting the appreciation of his or her professional performance, thus nurturing the instructor’s self-esteem. In fact, most of the instructors explicitly asked to review their group’s formal feedback forms, an indication of their perception that the group’s final feedback was an evaluation of their performance. Figure 4 illustrates the two sources that nurture instructors’ self-esteem.

### 3.8. Self-actualization

The analysis revealed that 21.5% (45 phrases) of the instructors’ reports dealt with issues related to self-actualization, which in MHN involves reaching one’s potential and desired goals. Two interrelated themes emerged at this level: *realization of the instructor’s role* and the *instructor’s experience of satisfaction.*

#### 3.8.1. Realization of instructor’s role

Interestingly, many instructors began their responses by referring to what they perceived as the learning outcomes.

There was meaningful learning … students said that they felt better prepared to face challenges in their practical work … I can clearly see that they leave the workshop better equipped for their jobs and I find this very fulfilling … at the end of the day, this is why I’m here (REF-W38).

Self-actualization occurs, according to Maslow (1943), when individuals realize their full potential, or when they attain their desired goals (Shaughnessy et al., 2018). Hence, given that the instructors’ role is to mediate the learning process, their need for self-actualization is gratified when such a process has successfully occurred. By the same token, when such a process is not achieved, instructors’ sense of self-realization is absent. Unsurprisingly, the achievement of their goals was significantly related to instructors’ overall experience of satisfaction.

#### 3.8.2. Instructors’ experience of satisfaction

When they were able to successfully realize their role as mediators of the SBL learning process, the emotions experienced by the instructors included enjoyment, reward, and a sense of satisfaction: ‘I felt that I had contributed to the participants’ professional development and that was so rewarding. I enjoyed it’ (ECO-47).

In contrast, when self-realization was not achieved, the emotions experienced included frustration, despair, disappointment, and sometimes bitterness. ‘Frankly, I am quite disappointed …. We did not gain much learning. So frustrating!!’ (ECO-56).

In conclusion, self-actualization consisted of two aspects: The first involved a *cognitive* process, during which instructors examined whether their ultimate goal of mediating the learning was achieved, whereas the second focused on their *emotional* experience as a result of this achievement or lack thereof.

### 3.9. The SBL process in light of instructors’ needs, as related to MHN – Two possible paths

The following section describes the process of a successfully completed SBL process vs. an incomplete SBL process, depending on whether the hierarchical needs were fully or partially met, from the instructors’ perspectives.

#### 3.9.1. The complete process, in which all needs are met

The analysis revealed that 62 of the 68 workshops reviewed in this study corresponded to the complete process. In this path, all five of the hierarchical levels of needs were addressed in the simulation workshop: a condition that enabled the instructor to achieve self-actualization. As we analyze Debra’s (all participant names are pseudonyms) description of her experience as an SBL workshop instructor, we find that her sense of self-actualization (level 5 need) includes the fulfilment of her needs from all of the preceding levels.

The simulations contributed a great deal [level 5–self-actualization]. The students explicitly expressed their enthusiasm [level 4–self-esteem]. I have been working with this group since the beginning of the academic year and there was a pleasant atmosphere [level 3–belonging].

As can be seen, no mention was made of first or second-level needs (neither basic nor security needs). Disregarding these first two levels was typical of cases in which a complete process had taken place, most likely because when fundamental needs are met, there is no need to mention them. However, when these two basic-level needs were not met, their absence was very conspicuous, as shown in the following section.

#### 3.9.2. The incomplete process, in which needs remain unmet

Six of the 68 workshops conducted corresponded to the path of ungratified needs. In these cases, the instructor reported that the goals of the SBL were not achieved and, to explain why, referred to one or more needs that remained unfulfilled throughout the workshop. Workshops that do not achieve their predefined goals constitute an important test case, through which lessons can be learned and improvements can be made. It is thus worthwhile to present the following two examples of workshops that were conducted by different instructors and involved different target populations. The analysis and MHN-related conceptualizations of the excerpted quotes regarding ungratified needs are presented in brackets.

Lea’s report, after conducting an SBL workshop for preservice teachers

The pages and forms were disorganized; the tablets didn’t work (at the last moment I had to photocopy pages) [level 1 – basic needs; equipment]. In general, I’m not sure whether we were able to delve deep enough or whether I allowed the group enough room for participants to express themselves [level 4 –self-esteem; self-reflection]. … When the students arrived, they were very tired and resistant, so they did not really cooperate. I didn’t feel comfortable in the group, I actually had to disarm their resistance [level 3 – belonging; dynamics during the workshop]. The group’s pedagogical mentor was present at the workshop and she felt it was important that she have her say. I asked her to enable the group members to express themselves [level 2 – security; authority figure]. But all in all, I’m not sure to what extent we were able to delve into the essential matters. Quite disappointing, I must admit [level 5 – self-actualization].

Naif’s report after conducting a workshop for a group of inservice teachers

From the beginning I felt that the relationships among the school staff were loaded and problematic, I found myself wondering how I should fit in [level 3 – belonging]. In the first simulated round, the participant could not be heard, due to a bug in the video system [level 1 – basic needs; workshop ingredients]. Throughout the workshop, the passive resistance was palpable and had a significant effect on the entire process [level 3 – belonging; group dynamics during the workshop]. In the second round, there were no volunteers. The vice-principal of the school was present and intervened, which in my view, prohibited an open discussion during the workshop [level 2 – security; authority figure]. My numerous attempts to break down participants’ resistance were only partially successful [level 4 –self-esteem; self-reflection]. What a waste of valuable resources!! [level 5 – self-actualization]

The SBL workshops that were characterized as ‘incomplete’ were described in greater detail (manifested in the length of the transcript text) than those characterized as ‘complete’ (shorter segments in the transcripts). Additionally, these longer descriptions tended to describe the process in a way that aligns with Maslow’s hierarchy. By providing these lengthy descriptions, the instructors inadvertently attributed the lack of completion of the SBL process to their needs that remained unmet.

## 4. Discussion

Using Maslow’s Hierarchy of Needs as a theoretical framework, the current study provides theoretical insight into the needs of SBL instructors. As these instructors are the ones mediating the learning process (Sellberg, 2018) by leading the debriefing – the most essential ingredient in SBL – it is imperative to examine their needs. Knowledge about SBL (particularly about instructors’ needs when leading SBL) in the field of teacher education is limited. As such, this study contributes to the existing literature by enhancing the theoretical understanding of instructors’ needs and highlighting the importance of meeting them. In turn, this information can be used to improve the quality of SBL-based teacher education. That is, the goal of the SBL in teacher education is to provide preservice and inservice teachers with the opportunity to analyze challenges that resemble those they encounter in the professional field; the likelihood of realizing this goal increases as more of the SBL instructors’ needs are met. Consequently, gaining an understanding and thus being able to fully address the SBL instructors’ needs is ultimately more beneficial for the teachers participating in the SBL workshops. To this end, practical recommendations for optimizing SBL instructors’ training and promoting SBL best practices can be formulated.

Overall, we were able to link instructors’ needs with the principles of Maslow’s theory (Maslow, 1943), which posits that self-actualization depends on meeting the needs of the hierarchy’s preceding levels. Accordingly, when instructors’ needs were gratified, they felt that they successfully fulfilled their role as instructor (i.e., mediating the learning), and they experienced satisfaction. Conversely, when instructors’ needs were not met, they felt frustrated and disappointed. In such cases, instructors’ reports included an elaborate explanation of why this ‘failure’ occurred, revealing that when lower-level needs were not met, then higher-level needs could not be met either, bearing out the principles underlying MHN (i.e., that self-actualization is achieved only when the needs of preceding levels are met). The theoretical insights and implications drawn from this study are presented and discussed according to the five levels of Maslow’s hierarchy.

### 4.1. Basic needs

The fact that certain needs (equipment, scenario, and actor) were mentioned early on in the instructors’ descriptions suggests that these were fundamental to the SBL instructors. Moreover, whenever these needs were not met, the instructors were concise in their comments and did not elaborate further about higher-level needs. These two features together indicate the fundamental nature of these needs, in line with MHN. Findings suggest that to meet instructors’ basic needs, the following three issues should be addressed: First, in accordance with scholars’ recent claims (Fisher and Royster, 2016), who argue that equipment and supplies are considered basic needs*, providing adequate equipment* is basic to instructors’ ability to manage the simulation. This finding aligns with Ferguson’s (2017) prior recommendations.

Second, designing *suitable scenarios* is imperative to instructors, as it creates a sense of authenticity that facilitates instructors’ role. Although authenticity was previously documented as crucial to *workshop participants*’ experience in SBL (Campbell and Daley, 2017), the current study highlights its importance for *instructors’* ability to lead SBL. In the same vein, as technology-related needs in this context coincide with Maslow’s basic needs (Bailey and Pownell, 1998), instructor training should include technology orientation, as previously argued (Fanning and Gaba, 2007).

Third, as the *workshop’s actors* bring the scenarios to life, employing professional actors is an essential basic requirement. The necessity of professional actors was previously advocated for the purposes of promoting authenticity (Pascucci et al., 2014), yet the current study highlights its necessity for instructors’ best functioning.

### 4.2. Security needs

Maslow’s theory was previously applied to explain the need for a safe learning environment in general (Shaughnessy et al., 2018), and particularly in the context of SBL *participants* (Tutticci et al., 2018). However, the current study underscores that security needs should be met not only for learners but also for *instructors*. Indeed, it was argued that instructors must be prepared to face unexpected challenging situations (Der-Sahakian et al., 2015). The current study exemplifies one of these challenges: the presence of an authority figure who may undermine the instructor. When the instructor feels threatened, the ability to function optimally is impaired, and it is likely that the workshop participants’ learning experience will likewise be suboptimal. This echoes previous claims that learners’ perceptions of teachers as authority figures may affect the teaching-learning process (Gil-Madrona et al., 2020). Hence, instructors should be provided with preliminary training focusing on how to handle such perceived threats in the context of leading a workshop to ensure that teachers receive the best SBL conditions.

### 4.3. Belongingness needs

Corresponding to Maslow’s sense of belonging, which includes the desire for collaboration and harmonious existence with others, establishing a sense of belongingness with the group was experienced by instructors as a vital need, allowing them to perform optimally. This finding echoes a previous study that found that an important factor that influenced teacher mentors’ self-actualization was the relationship that developed during the mentoring process (Fletcher, 2006). Our study further expands these findings by emphasizing the importance of relationships in the context of SBL instruction as well. In fact, the sense of belonging is particularly relevant as SBL involves group peer learning (Levin and Flavian, 2020). Hence, it may be challenged in cases of instructor-group diversity. Instructor training should therefore aim to instil cultural sensitivity and social justice norms (Grogan, 2014; Martinek et al., 2021; Losada Puente et al., 2022), to bridge potential gaps. Noteworthy, intercultural competence was previously explored as an outcome variable of SBL (Harder, 2018), yet this study highlights the need to view cultural differences as a factor that affects instructors’ ability to function effectively. Training instructors to speak the group’s cultural ‘lingo’ is vital to instructors’ belongingness needs and overall performance.

### 4.4. Self-esteem needs

In accordance with MHN’s fourth level, which involves the need to be competent and to achieve, as well as to be recognized and appreciated (Maslow, 1943 in Adams et al., 2015), instructors reported engaging in a dual process of reflection, based on two sources: instructors’ *internal* self-reflection (about their competence and achievements) and the perceived feedback from the group (i.e., *external* recognition and appreciation).

Whereas prior SBL research has demonstrated that reflection promotes effective learning among *participants* (Butvilofsky et al., 2012), the current study underscores the importance of reflection for addressing *instructors’* needs during SBL. Thus, allocating time to instructors for receiving participants’ reflections, granting them access to feedback questionnaires, encouraging them to engage in self-reflection, and using peers’ reflective discourse (Der-Sahakian et al., 2015) may all be channels through which instructors’ need for self-evaluation (as part of their self-esteem needs) is addressed.

### 4.5. Self-actualization

The peak of MHN involves reaching self-actualization, which essentially involves the realization of one’s desired goals (Shaughnessy et al., 2018) and occurs when individuals realize their full potential (Maslow, 1943). Instructors’ reports indicated two components of self-actualization in the context of SBL: (a) Instructors’ actualization of their job-related potential as mediators of learning, manifested in the achievement of learning outcomes, and (b) The experience of satisfaction once instructors’ goals are achieved.

Findings further showed two possible paths that the SBL process can take. As expected, and in accordance with Maslow’s principles: (1) the self-actualization of instructors requires the meeting of all prior needs in the hierarchy, and (2) conversely, instructors’ unmet needs hinder their self-actualization and result in experiences of frustration and disappointment. Hence, it is essential to design workshops in a manner that ensures that all instructors’ needs are met, to optimize the likelihood that they will realize the workshop’s goals.

## 5. Implications and limitations

The study’s major implication for teacher education lies in the focus on the instructors’ needs, given that they are the ones who facilitate teachers’ learning through simulation. To date, the literature on educational simulations has focused solely on the workshop *participants*’ needs (Tutticci et al., 2018). The current study, however, sheds light on the *instructors’* needs and the components that may promote effective SBL from the instructors’ perspectives. In fact, instructors’ needs may be viewed as an indirect factor that ultimately affects the quality of teachers’ SBL experience. As such, the study contributes to the ongoing conversation regarding the ways in which learning through simulation can be understood and improved to promote teachers’ learning. The study has a few limitations. First, it is possible that instructors’ reflections were influenced by response biases. To minimize this possibility, a triangulation of research sources was established. Second, as the sample comprised mainly females, transferability may be limited. However, given the gender imbalance in the field of teacher education (Robinson et al., 2017), we believe that the sample is adequate. Third, the research was conducted in one specific teacher-education simulation center, which is located at a teacher-education college. Thus, transferability to teachers attending other colleges may be limited. As such, a multisite investigation of this issue is needed to further examine the research findings. Furthermore, given that this is a case study, transferring and comparing these findings to findings of other studies should be conducted with extra caution (Stake, 2013). For example, this study involved clinical simulations and, therefore, applying its findings about the SBL instructors’ needs to virtual simulations requires careful consideration. Consequently, additional studies are needed, to expand the paradigm to a multiple-case study. In addition, we call for a longitudinal examination of instructors’ needs, to determine whether these needs change over time. Finally, to better understand the impact of an authority figure’s presence during SBL, further research is recommended.

## 6. Conclusion

As instructors play a significant role in SBL, the current study contributes to the existing literature of teacher education by providing an in-depth theoretical conceptualization of SBL instructors’ needs and highlighting the importance of addressing them, to provide teachers with the best possible learning conditions. Addressing the study’s goal by demonstrating the SBL instructors’ needs vis-à-vis MHN enhances the theoretical understanding of this phenomenon and provides applicable insights that can be assimilated in SBL instructors’ training. Specifically, efforts should be made to address instructors’ varied needs, as follows: *basic needs* may be attended to by providing adequate equipment, designing suitable and authentic scenarios, and employing professional actors; *security needs* may be met by preparing instructors to face unexpected challenging situations and by providing preliminary training focused on handling perceived sources of threats when leading a workshop; to create a *sense of belonging*, instructors should receive cultural-competence training, to learn of ways to bridge potential gaps; *self-esteem* needs may be addressed by allocating time for receiving participants’ reflections and encouraging instructors to engage in self-reflection as part of their need for self-evaluation. Ultimately, all of these are ways to help instructors experience *self-actualization* and satisfaction. Given that SBL is becoming an integral part of teacher education and considering the essential role of the instructors in facilitating teachers’ learning through simulation, understanding how to address instructors’ needs and how to design optimal training for SBL instructors in teacher education is imperative. Future studies may opt to focus on instructors’ needs in the context of virtual simulations to further broaden our understanding of whether and/or how instructors’ needs are manifested differently in this context. To this end, studies should be conducted in a range of contexts, for example, in a multicultural framework, and with different participant populations. Furthermore, future studies should aim to explore whether these needs are shared across different learning environments or whether these are unique to SBL. To conclude, the current study underscores the importance of attending to instructors’ needs, to ensure that they can use the simulation tool in teacher education to its full potential.

## Data availability statement

The original contributions presented in the study are included in the article/Supplementary material, further inquiries can be directed to the corresponding author.

## Ethics statement

The studies involving human participants were reviewed and approved by Achva academic college ethics committee. The patients/participants provided their written informed consent to participate in this study.

## Author contributions

All authors listed have made a substantial, direct, and intellectual contribution to the work and approved it for publication.

## Conflict of interest

The authors declare that the research was conducted in the absence of any commercial or financial relationships that could be construed as a potential conflict of interest.

## Publisher’s note

All claims expressed in this article are solely those of the authors and do not necessarily represent those of their affiliated organizations, or those of the publisher, the editors and the reviewers. Any product that may be evaluated in this article, or claim that may be made by its manufacturer, is not guaranteed or endorsed by the publisher.
